# Prognostic significance of histological invasion in high grade soft tissue sarcomas

**DOI:** 10.1186/2193-1801-3-544

**Published:** 2014-09-22

**Authors:** Satoshi Tsukushi, Yoshihiro Nishida, Hiroshi Urakawa, Eiji Kozawa, Naoki Ishiguro

**Affiliations:** Department of Orthopaedic Surgery, Nagoya University Graduate School of Medicine, 65 Tsurumai-cho, Showa-ku, Nagoya, 466-8550 Japan

**Keywords:** Soft tissue sarcoma, Histological invasion, Prognosis, Clinical outcome

## Abstract

High grade soft tissue sarcomas often show histological invasion to adjacent compartment including bone and vessel. This study aimed to evaluate histological invasion in high grade soft tissue sarcomas, clarify its impact on prognosis and devise treatment strategies. We retrospectively reviewed 133 patients with non-small round cell high grade soft tissue sarcomas surgically treated between 2001 and 2011. Clinical and histological factors examined for prognostic value included age, gender, size, depth, location, adjuvant therapy and invasion to adjacent compartment. Study endpoints included overall survival rate, event free survival rate and local recurrence free survival rate, estimated by the Kaplan-Meier method. Univariate and multivariate analyses were performed using the log-rank test and Cox proportional hazards model. Local recurrence was recognized in 14 cases (11%). The 5-year overall survival rate and 5-year event free survival rate were 82.2% and 63.6% respectively. The metastasis-free survival rate at 5 years and local recurrence-free survival rate at 5 years were 69.8% and 86.8% respectively. Histological invasion to adjacent compartment was noted in 52 cases (39%), and was significantly correlated with histological type (p = 0.01), tumor size (p = 0.009), and depth (p < 0.05). In multivariate analyses, we showed that tumor size and histological invasion were significant independent predictors of OS (hazard ratio 8.1/2.5) and EFS (hazard ratio 5.5/2.2), while only tumor size was a significant independent predictor of LRFS (hazard ratio 4.0). We evaluated the relation between histological invasion and the oncological outcomes of high grade soft tissue sarcomas. In multivariate analyses, histological invasion was found to be an independent adverse prognostic factor with hazard ratios of 2.2-2.5, suggesting that a detailed assessment of these factors is essential. Histological invasion showed a correlation with tumor size and histological type, and the surgical margin should be decided based on these factors.

## Introduction

Soft tissue sarcoma is an uncommon cancer with variable histological subtypes and clinical behaviors. Once metastases develop most patients die of their disease. Numerous studies have identified clinical prognostic factors that could influence survival and local recurrence of soft tissue sarcoma (Behranwala et al. 2004; Carneiro et al. 2011; Coindre et al. 1996; Collin et al. 1987; Eilber et al. 2004; Eilber et al. 2003; Gaynor et al. 1992; Heise et al. 1986; Kattan et al. 2002; Lahat et al. 2008; McKee et al. 2004; Mutter et al. 2012; Pisters et al. 1996; Ramanathan et al. 1999; Riad et al. 2004; Rööser et al. 1988; Singer et al. 1994; Stefanovski et al. 2002; Stojadinovic et al. 2002; Stotter et al. 1990; Trojani et al. 1984; Ueda et al. 1988; Zagars et al. 2003). It would be beneficial for clinical management and research to identify these factors. However soft tissue sarcomas represent a heterogeneous group of rare malignant tumors with a wide spectrum of histological grade. There are few large studies that have focused on high grade sarcomas. Most of soft tissue sarcomas characteristically show histological invasion to adjacent tissues. This makes local control more difficult, and may necessitate wide resection including adequate amounts of normal tissue. Especially high grade sarcomas, often extend beyond their compartment of origin, invading adjacent compartments, and showing microscopic invasion to bone and neurovascular bundles. These features may not only complicate or preclude limb-preserving surgery but also provide distant metastasis in the early period, markedly worsening the prognosis (Ferguson et al. 2006; Merimsky et al. 1998; LeVay et al. 1993). It is therefore important to recognize to what extent this kind of histological invasion is detected in high grade sarcomas, and what influence it exerts on survival and local recurrence.

This study was undertaken to assess histological invasion of high grade sarcomas to adjacent compartments, define its influence on prognosis, and suggest more suitable therapeutic strategies on the basis of the results obtained.

## Materials and methods

The prospectively collected database was retroepectively reviewed to identify all patient with non metastatic non-small round cell soft tissue sarcomas surgically treated between 2001 and 2011 in our institution. We identified 183 patients with non-small round cell soft tissue sarcomas. We excludes 50 low grade sarcomas (20 dermatofibrosarcoma protuberans, 13 well differentiated liposarcomas, 7 solitary fibrous tumors, 6 low grade fibromyxoid sarcomas and 4 low grade fibrosarcoma). The remaining 133 patients (78 males, 55 females) were studied. Clinical and histological factors examined for prognostic value included age, gender, size, depth, location, adjuvant therapy and invasion to adjacent compartments. Study endpoints included overall survival rate (OS), event free survival rate (EFS) and local recurrence free survival rate (LRFS), estimated by the Kaplan-Meier method. Univariate and multivariate analyses were performed using the log-rank test and Cox proportional hazards model.

In all cases histological invasion to adjacent compartments was evaluated in detail in resected specimens, and classified as follows: (1) extra-compartmental invasion by intramuscular or intermuscular tumor destroying the adjacent fascia; (2) superficial tumor destroying the deep level of the fascia and invading the deep level of the muscle; (3) tumor destroying the adjacent periosteum or bone; (4) invasion of adjacent vascular sheaths or nerve sheaths.

Tumor size was measured on preoperative images obtained by MRI and other imaging modalities. Tumors were initially classified histologically into low or high grade according to the classification of Broders et al. (Grades 1 and 2 as low and grades 3-4 as high grade) and in recent years according to the French Federation classification (FNCLCC) (grade1 as low grade and grades 2 and 3 as high grade).

Tumor size was measured on preoperative MRI and other images. Histopathological measurements were made in cases resected at the prereferral hospital. The patients’ clinicopathological data are listed in Table 1.

**Table 1:** **Clinicopathological data for 133 patients with non-small round cell high grade soft tissue sarcomas**

Characteristics	All	%	With invasion	Without invasion	P-value**
	(N = 133)		(N = 52)	(N = 81)	
Age					
Median	62 yr				
<60 yr	56	42	16	38	
≧60 yr	77	58	36	43	p = 0.064
Gender					
Male	78	59	32	46	p = 0.587
Female	55	41	20	35	
Tumor location					
Upper extremity	23	17	11	12	
Lower extremity	85	64	28	56	
Trunk	25	19	13	12	p = 0.142
Tumor depth					
Superficial	62	47	13	49	
Deep	71	53	39	32	p < 0.05
Tumor size					
≦5 cm	46	35	11	35	
>5 cm	87	65	41	46	p = 0.009
Surgical margin					
R0	122	92	39	74	
R1	11	8	13	7	p < 0.05
R2	0	0	0	0	
Histological diagnosis					
Undifferentiated pleomorphic sarcoma	52	39	31	21	p = 0.01
Liposarcoma	23	17	4	19	
Myxofibrosarcoma	14	11	3	11	
MPNST*	14	11	5	9	
Synovial sarcoma	13	10	4	9	
Leiomyosarcoma	10	8	3	7	
Other	7	5	2	5	
Continuously free of disease	82	62	22	60	
Alive with disease	8	6	4	4	
Alive and currently no evidence of disease	18	14	10	8	
Dead of disease	22	17	14	8	
Dead of other disease	3	2	2	1	
Overall survival rate at 5 years	82.2%		69.6%	90.3%	
Event-free survival rate at 5 years	63.6%		41.3%	77.5%	
Metastasis-free survival rate at 5 years	69.8%		53.0%	80.8%	
Local recurrence-free rate at 5 years	86.8%		77.8%	92.1%	

*MPNST; malignant peripheral nerve sheath tumor.

**The risk factor of invasion was assessed using Fisher's exact test or the Chi-square test.

In the case of soft tissue sarcomas we aim for a wide resection securing a 3 cm margin from the tumor edge. In cases subjected to an unplanned excision we undertake definitive surgery within 2 months of the unplanned procedure to secure a 3 cm margin from the site of the previous surgical manipulations. In all cases a detailed postoperative histological assessment was made of the resected specimen. Resections were defined as R2 if the margins were macroscopically positive, R1 if all macroscopic disease was removed but with the margins microscopically positive, or R0 if margins were microscopically negative. Postoperative radiotherapy was administered to selected cases of R0 with a close margin, R1and R2.

All patients were followed by physical examination at every visit to detect any local recurrence, MRI of the primary site every 6 months, and CT of the chest after surgical treatment every 3 months until 2 years after surgery and every 6 months thereafter.

Age ranged from 8 to 91 years (mean 62 years), The minimum duration of follow-up was 12 months (mean, 51 months; range 12 ~ 134 months). Site of origin was the upper extremities in 23 cases, lower extremities in 85, and trunk in 25. Tumor localization was superficial in 62 cases, and deep in 71. The pathological diagnosis was undifferentiated pleomorphic sarcoma in 52 cases, liposarcoma in 23, myxofibrosarcoma in 14, malignant peripheral nerve sheath tumor in 14, synovial sarcoma in 13, leiomyosarcoma in 10, and other types in 7.

### Statistical analyses

Survival was estimated by the Kaplan-Meier method. OS, EFS and LRFS were compared with the log-rank test. The multivariate analysis was performed using a Cox proportional hazard model. The variables included in the multivariate analysis were the significant factors identified in the univariate analysis. The risk factor of histological invasion was assessed using Fisher's exact test or the Chi-square test. In all statistical analyses, p < 0.05 was considered to be significant. Statistical analysis was performed using SPSS Version 21.0 (SPSS Inc, Chicago, IL).

## Results

The oncological outcome was continuous disease free in 82 cases, no evidence of disease in 18, alive with disease in 8, dead of disease in 22, and dead of other disease in 3. Local recurrence was recognized in 14 cases (11%). The 5-year cumulative survival and 5-year disease-free survival rates were 82.2% and 63.6% respectively (Table 1). The metastasis-free survival rate at 5 years and local recurrence-free survival rate at 5 years were 69.8% and 86.8% respectively.

The overall survival rate at 5 years and the event free survival rate at 5 years in the cases without invasion were 90.3% and 77.5% respectively. On the other hand, in the cases with histological invasion to an adjacent compartment the overall survival rate at 5 years and the event free survival rate at 5 years were much lower at 69.6% and 41.3% respectively.

After definitive surgery, 122 patients (92%) had R0 margins and 11 (8%) R1 margins. Thirty-two cases (24%) underwent postoperative radiotherapy. Chemotherapy at the time of the initial treatment was administered to 28 cases (21%) including 13 of synovial sarcoma.

**Figure 1:**
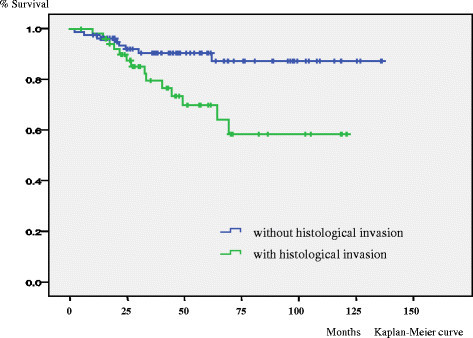
**Kaplan–Meier life table analysis of overall survival of high grade soft tissue sarcoma patients with or without histological invasion.** For overall survival histological invasion (p = 0.01) was a significant unfavorable prognostic factor.

**Figure 2:**
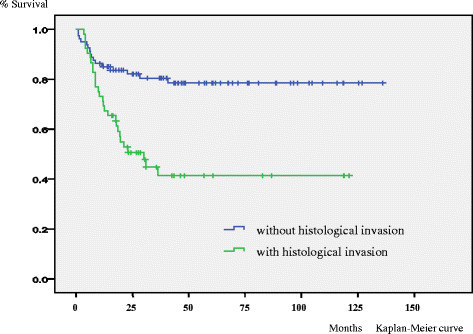
**Kaplan–Meier life table analysis of event free survival of high grade soft tissue sarcoma patients with or without histological invasion.** For event free survival histological invasion (p < 0.001) was a significant unfavorable prognostic factor.

**Figure 3:**
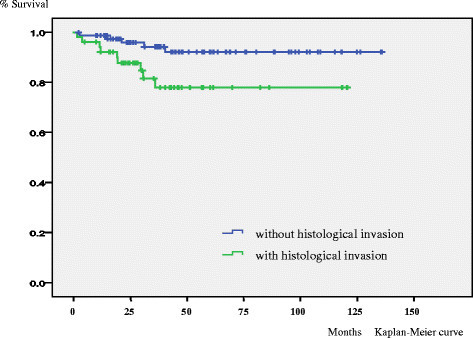
**Kaplan–Meier life table analysis of local recurrence free survival of high grade soft tissue sarcoma patients with or without histological invasion.** For local recurrence free survival histological invasion (p = 0.025) was a significant unfavorable prognostic factor.

In the univariate analysis size (p < 0.001), depth (p < 0.001) and histological invasion (p = 0.01) were significant unfavorable prognostic factors for overall survival. (Figure 1) Size (p < 0.001), depth (p < 0.001) and histological invasion (p < 0.001) were significant unfavorable prognostic factors for event free survival. (Figure 2) Gender (p < 0.05), size (p < 0.01), depth (p < 0.01) and histological invasion (p = 0.025) were significant unfavorable prognostic factors for local recurrence free survival. (Figure 3) Univariate analyses for prognostic factors are listed in Table 2.

**Table 2:** **Univariate analysis for prognostic factors in overall survival, event free survival and local recurrence free survival**

Factors	No. of patients	Overall 5-year survival rate	P-value	Event free 5-year survival rate	P-value	Local recurrence free 5-year survival rate	P-value
Age							
≧60 yr	56	80.8%	p = 0.298	64.4%	p = 0.711	87.0%	p = 0.926
<60 yr	77	84.5%		62.0%		86.5%	
Gender							
Male	78	78.7%	p = 0.362	55.4%	p = 0.067	96.3%	p = 0.040 *
Female	55	86.9%		75.8%		80.5%	
Tumor location							
Extremity	108	83.4%	p = 0.271	64.9%	p = 0.874	89.7%	p = 0.075
Trunk	25	76.6%		58.3%		74.4%	
Tumor depth							
Superficial	62	94.2%	p < 0.001*	82.3%	p < 0.001*	95.7%	p < 0.01*
Deep	71	71.0%		45.7%		77.4%	
Tumor size							
≦5 cm	46	100.0%	p < 0.001*	90.8%	p < 0.001*	100.0%	p < 0.01*
>5 cm	87	71.6%		48.1%		78.1%	
Histological invasion							
Yes	52	69.6%	p = 0.01*	41.3%	p < 0.001*	77.8%	p = 0.025*
No	81	90.3%		77.5%		92.1%	
Adjuvant radiotherapy							
Yes	32	76.5%	p = 0.964	57.1%	p = 0.538	78.7%	p = 0.128
No	101	84.7%		66.1%		90.1%	
Adjuvant chemotherapy							
Yes	28	88.0%	p = 0.596	66.2%	p = 0.952	91.8%	p = 0.625
No	105	81.3%		63.4%		86.0%	

*Statistically significant difference.

In the multivariate analysis, size (hazard ratio 8.1) and histological invasion (hazard ratio 2.5) were the independent factors associated with overall survival. Size (hazard ratio 5.5) and histological invasion (hazard ratio 2.2) were the independent factors associated with event free survival, while size alone (hazard ratio 4.0) was associated with local recurrence free survival. Multivariate analyses for prognostic factors are listed in Table 3.

**Table 3:** **Multivariate cox proportional analysis for prognostic factors in overall survival, event free survival and local recurrence free survival**

Factors	Overall survival		Event free survival		Local recurrence free survival	
	Hazard ratio (95% CI)	P-value	Hazard ratio (95% CI)	P-value	Hazard ratio (95% CI)	P-value
Age						
≧60 yr					1 (referent)	0.959
<60 yr					1.0 (0.33-2.79)	
Tumor depth						
Superficial	1 (referent)	0.961	1 (referent)	0.228	1 (referent)	0.863
Deep	1.0 (0.39-2.35)		1.5 (0.78-2.81)		1.1 (0.35-3.47)	
Tumor size						
≦5 cm	1 (referent)	0.015*	1 (referent)	0.002*	1 (referent)	0.006*
>5 cm	8.1 (2.46-26.42)		5.5 (1.90-15.75)		4.0 (1.38-10.13)	
Histological invasion						
No	1 (referent)	0.036*	1 (referent)	0.012*	1 (referent)	0.08
Yes	2.5 (1.35-4.71)		2.2 (1.19-4.16)		1.8 (1.11-3.15)	

*Statistically significant difference.

In 52 cases (39%) histological invasion of an adjacent compartment was detected. Histological invasion was significantly associated with size (p = 0.009), depth (p < 0.05) and histological subtype (undifferentiated pleomorphic sarcoma: p = 0.01) (Table 1).

## Discussion

Soft tissue sarcoma is an uncommon cancer with variable histological subtypes and clinical behaviors. Once metastases develop most patients die of their disease. Numerous studies have identified clinical prognostic factors that could influence survival and local recurrence of soft tissue sarcoma (Behranwala et al. 2004; Carneiro et al. 2011; Coindre et al. 1996; Collin et al. 1987; Eilber et al. 2004; Eilber et al. 2003; Gaynor et al. 1992; Heise et al. 1986; Kattan et al. 2002; Lahat et al. 2008; McKee et al. 2004; Mutter et al. 2012; Pisters et al. 1996; Ramanathan et al. 1999; Riad et al. 2004; Rööser et al. 1988; Singer et al. 1994; Stefanovski et al. 2002; Stojadinovic et al. 2002; Stotter et al. 1990; Trojani et al. 1984; Ueda et al. 1988; Zagars et al. 2003). Soft tissue sarcomas are characterized by aggressive histological invasiveness, which has been correlated with their prognosis (Gustafson et al. 2003; Carneiro et al. 2011). Almost all high grade sarcomas show histological extracapsular invasion. Meanwhile, myxofibrosarcomas whose local control is especially difficult because of aggressive invasiveness have been reported to develop significantly fewer distant metastases than leiomyosarcomas (Mutter et al. 2012). In the present subgroup analysis as well, although almost all myxofibrosarcomas showed an invasive growth pattern the prognosis was favorable provided that local control was achieved. On the other hand, the invasive growth patterns can be divided into two groups. One is the intra-compartmental invasion limited to within the compartment of tumor origin which is frequently observed in myxofibrosarcomas. The other is the extra-compartmental invasion which extends beyond the compartment to involve an adjacent compartment. The latter in our experience often assume a more aggressive clinical course. This prompted us to undertake the present study to determine whether cases with this kind of histological extra-compartmental invasion show differences in metastases, survival, and local recurrence as compared with those without it. In high grade sarcomas, histological invasion to adjacent compartments was found to be a significantly unfavorable prognostic factor as compared to the absence of invasion with regard to overall survival, event free survival, and local recurrence free survival. In the multivariate analysis as well, in addition to size, histological invasion was noted to be an independent unfavorable prognostic factor for both overall survival and event free survival.

With extensive discussion between surgeons and pathologists, as many sections as possible are evaluated. However, it may still be very difficult to fully evaluate the invasion status particularly in larger specimens. For this reason, our study is thus limited by the fact that microscopic invasion may have been overlooked to some extent by pathology. The prognosis of deep soft tissue sarcomas showing histological invasion of bone and vascular bundles has been reported to be poor (Ferguson et al. 2006; Merimsky et al. 1998; LeVay et al. 1993). This means that these tumors have a propensity to show histological invasion beyond their compartment of origin. Considering the present results in which histological invasion was demonstrated to exert an adverse influence on prognosis, more detailed and accurate histological assessment will be required to devise more appropriate therapeutic strategies. For high grade sarcomas, the establishment of new stratified therapies taking into account this factor is urgently needed.

In the multivariate analysis in this study, invasion did not reach significance as an independent unfavorable prognostic factor for local recurrence-free survival, although our experience suggests that in cases showing invasion the local recurrence rate is high. Wide resection of a tumor limited to a single compartment is technically relatively simple, whereas once a tumor extends beyond its compartment of origin it becomes technically demanding to achieve a wide resection with adequate margin. The results of the present prognostically unfavorable cases may have been biased by the inclusion of cases in which there was a competition between local recurrence and death. We thereby might underestimate the local recurrence rate. Histological invasion was significantly correlated with larger size (p = 0.009), deep location (p < 0.05) and undifferentiated pleomorphic sarcoma histological subtype (p = 0.01). For cases with such factors we recommend that more aggressive surgery be provided and that the indications for adjuvant radiotherapy be liberally expanded.

In conclusion, high grade soft tissue sarcomas often show an extra-compartmental invasive growth pattern with involvement of bone, blood vessels, and other structures. This not only makes local control much more difficult to achieve, but in the present multivariate analysis was demonstrated to be an independent unfavorable prognostic factor for both overall survival and event free survival. Further investigations to evaluate invasiveness that cannot be evaluated by the histological grade alone and to apply stratified therapy based on the obtained results are needed.
